# INTERVENTION WITH RAPAMYCIN TO IMPROVE HEALTHY AGING AND LONGEVITY IN A NON-HUMAN PRIMATE

**DOI:** 10.1093/geroni/igz038.403

**Published:** 2019-11-08

**Authors:** Adam B Salmon

**Affiliations:** University of Texas Health at San Antonio, San Antonio, Texas, United States

## Abstract

Interventions to extend lifespan and improve health with increasing age will have significant impact on a growing aged population. Several pharmaceutical interventions extend lifespan in laboratory rodent models with rapamycin, an inhibitor of mechanistic target of rapamycin (mTOR) being the most well studied. Bridging towards translation, we have an ongoing long-term study testing whether rapamycin treatment can extend lifespan and delay the progression of age-related disease in a short-lived non-human primate species, the common marmoset (Callithrix jacchus). We show that daily oral dosing of slow-releasing, encapsulated rapamycin will result in clinically effective concentrations of rapamycin in the blood and inhibit mTOR signaling. This treatment is well tolerated and does not dramatically promote known side effects of this drug, including altering clinical hematology, immune cell subsets, or promoting metabolic dysfunction including glucose intolerance in comparison to control aging marmosets. Unlike previous reports in rodents, rapamycin does not have clear effects on aging cardiovascular function in marmosets. However, in our oldest cohorts daily rapamycin treatment tends to prevent age-associated changes in body mass and composition and prevent decline in kidney function. Now more than three years after beginning treatment, we are now starting to assess the effects of rapamycin on marmoset longevity. When complete, this study will describe for the first time the potential for pharmaceutical intervention to extend longevity of a primate species with the ultimate goal of significant translational impact to human aging.

